# Defective ryanodine receptor N-terminus inter-subunit interaction is a common mechanism in neuromuscular and cardiac disorders

**DOI:** 10.3389/fphys.2022.1032132

**Published:** 2022-10-12

**Authors:** Yadan Zhang, Camille Rabesahala de Meritens, Astrid Beckmann, F. Anthony Lai, Spyros Zissimopoulos

**Affiliations:** ^1^ Institute of Life Science, Swansea University Medical School, Swansea, United Kingdom; ^2^ College of Medicine and Biomedical Research Centre, Qatar University, Doha, Qatar

**Keywords:** amino-terminus, catecholaminergic polymorphic ventricular tachycardia, malignant hyperthermia, inter-subunit interaction, tetramerization, ryanodine receptor (RyR)

## Abstract

The ryanodine receptor (RyR) is a homotetrameric channel mediating sarcoplasmic reticulum Ca^2+^ release required for skeletal and cardiac muscle contraction. Mutations in RyR1 and RyR2 lead to life-threatening malignant hyperthermia episodes and ventricular tachycardia, respectively. In this brief report, we use chemical cross-linking to demonstrate that pathogenic RyR1 R163C and RyR2 R169Q mutations reduce N-terminus domain (NTD) tetramerization. Introduction of positively-charged residues (Q168R, M399R) in the NTD-NTD inter-subunit interface normalizes RyR2-R169Q NTD tetramerization. These results indicate that perturbation of NTD-NTD inter-subunit interactions is an underlying molecular mechanism in both RyR1 and RyR2 pathophysiology. Importantly, our data provide proof of concept that stabilization of this critical RyR1/2 structure-function parameter offers clear therapeutic potential.

## Introduction

Myocyte contraction is effected by a transient rise in the intracellular Ca^2+^ concentration mediated by the ryanodine receptor (RyR), the Ca^2+^ release channel of the sarcoplasmic reticulum. It is triggered by an action potential that depolarizes the plasma membrane to activate the voltage-gated Ca^2+^ channel, also known as dihydropyridine receptor (DHPR). In adult mammalian heart, the ensuing small Ca^2+^ influx activates the RyR to result in a much larger Ca^2+^ release from the sarcoplasmic reticulum Ca^2+^ store (Bers, 2002). This process, termed Ca^2+^-induced Ca^2+^ release, is also operational in skeletal muscle, but its contribution to contraction of adult mammalian muscles appears negligible (Rios, 2018). Unlike the heart, skeletal muscle is able to contract in the absence of extracellular Ca^2+^ influx due to physical coupling between DHPR and RyR, where the action potential-evoked conformation change in DHPR is mechanically transmitted to open the RyR (Hernandez-Ochoa and Schneider, 2018). The RyR, a homotetramer of ∼2.2 MDa, is the largest ion channel known to exist (Zissimopoulos and Lai, 2007). There are three mammalian isoforms, RyR1, the predominant type in skeletal muscle, RyR2, the exclusive type in cardiac muscle, and RyR3, which is expressed at low levels in skeletal and smooth muscles. The three RyR isoforms have ∼70% peptide sequence homology and very similar geometry.

Abnormal SR Ca^2+^ release due to missense mutations in *RYR1* and *RYR2* results in neuromuscular (e.g., malignant hyperthermia, MH) and cardiac disease (e.g., catecholaminergic polymorphic ventricular tachycardia, CPVT), respectively (Hernandez-Ochoa et al., 2016; Fowler and Zissimopoulos, 2022). Mutations are found throughout the RyR peptide sequence, but tend to concentrate on five structural domains, namely, the N-terminus domain (NTD), bridging solenoid B, core solenoid, transmembrane domain and C-terminus domain (Fowler and Zissimopoulos, 2022). Neighboring NTDs from the four RyR subunits interact with each other to promote channel closure (Tung et al., 2010; Zissimopoulos et al., 2013; Zissimopoulos et al., 2014). Notably, pathogenic RyR1 and RyR2 mutations impair NTD-NTD inter-subunit interactions and result in hyperactive and leaky channels (Iyer et al., 2020; Seidel et al., 2021; Seidel et al., 2015; Xiong et al., 2018; Zheng and Liu, 2017).

The aim of this study was two-fold. First, to empirically assess whether defective RyR1 NTD tetramerization is involved in MH. Second, to explore targeted amino acid modification for potential to restore RyR2 NTD tetramerization in CPVT.

## Materials and methods

### Materials

The human embryonic kidney (HEK) 293 cell line was obtained from ATCC^®^ (CRL-1573), mammalian cell culture reagents from Thermo Scientific, electrophoresis equipment and reagents from Bio-Rad, enhanced chemiluminescence detection kit from Thermo Scientific, mouse anti-cMyc (9E10) from Santa Cruz Biotechnology, goat anti-mouse IgG conjugated with horseradish peroxidase from Abcam, DNA restriction endonucleases from New England Biolabs, Pfu DNA polymerase from Promega, site-directed mutagenesis kit (QuikChange II XL) from Agilent Technologies, oligonucleotides and all other reagents from Sigma.

### Plasmid construction

The plasmids encoding for wild-type rabbit RyR1 and human RyR2 N-terminal constructs tagged with the cMyc epitope at the N-terminus have been described previously (Zissimopoulos et al., 2014; Zissimopoulos et al., 2013). Desired missense mutations were generated using the site-directed mutagenesis QuikChange II XL kit and appropriate primers as recommended by the supplier. All plasmid constructs were verified by direct DNA sequencing.

### Chemical cross-linking

HEK293 cells were transiently transfected using TurboFect according to the provider’s instructions. 24 h post-transfection, cells were homogenized on ice in homogenization buffer (5 mM HEPES, 0.3 M sucrose, 10 mM DTT, pH 7.4) by 20 passages through a needle (0.6 × 30 mm) and dispersing the cell suspension through half volume of glass beads (425–600 microns). Cell homogenate free of nuclei and heavy protein aggregates was obtained by centrifugation at 1,500 *g* for 5 min at 4°C, followed by a second centrifugation step at 20,000 x *g* for 10 min at 4°C. Cell homogenate supernatant (20 μg) was incubated with glutaraldehyde (0.0025% or 260 μmol/L) for the following time-points: 0, 2, 5, 10, 15, 20, 30 and 60 min. The reaction was stopped with the addition of hydrazine (2%) and SDS-PAGE loading buffer (60 mM Tris, 2% SDS, 10% glycerol, 5 mM EDTA, 0.01% bromophenol blue, pH 6.8). Samples were analyzed by SDS-PAGE and Western blotting with Ab^cMyc^ (1:1,000 dilution). Tetramer to monomer ratio was determined by densitometry using a GS-900 Scanner (Bio-Rad) and Image Lab software (Bio-Rad). Tetramer formation was calculated as follows: T = OD_T_/(OD_T_ + OD_M_)x100, where OD_T_ and OD_M_ correspond to optical density obtained for tetramer and monomer bands respectively. Statistical analysis was carried out with GraphPad Prism software.

## Results

### MH mutation R163C disrupts RyR1 NTD tetramerization

We have previously shown that the CPVT R176Q mutation disrupts NTD tetramerization to produce a hyperactive channel (Seidel et al., 2021). The equivalent residue in human RyR1, R163, is frequently mutated in individuals susceptible to MH. Two different substitutions have been reported, R163C and R163L (Table 1). We chose to study the R163C variant because its gain of function characteristics have been studied extensively *in vivo* and *in vitro* (Tong et al., 1997; Avila and Dirksen, 2001; Yang et al., 2003; Yang et al., 2006; Feng et al., 2011; Chen et al., 2018; Iyer et al., 2020).

**TABLE 1:** Evidence for defective N-terminal inter-subunit interactions due to RyR1 (top) and RyR2 (bottom) pathogenic mutations within the β8-β9 and β23-β24 loops (ARVD2: arrhythmogenic right ventricular dysplasia 2, CCD: central core disease, CPVT: catecholaminergic polymorphic ventricular tachycardia, MH: malignant hyperthermia, SUD: sudden unexplained death).

RyR1/2 mutation	Disease	First report	Defective N-terminal inter-subunit interaction	References
Q155K	MH	Robinson *et al.*, Hum Mutat, 2006		
R156K	MH	Galli *et al.*, Hum Mutat, 2006		
R156W	Myopathy	Amburgey *et al.*, Orphanet J Rare Dis, 2013		
G159E	Myopathy	Witting *et al.*, Neurol Genet, 2017		
E160G	CCD	Shepherd *et al.*, J Med Genet, 2004		
R163C	CCD & MH	Quane *et al.*, Nat Genet, 1993	Cryo-electron microscopy	Iyer *et al.*, Sci Adv, 2020
			Chemical cross-linking	Present study
R163L	MH	Monnier *et al.*, Hum Mutat, 2005		
G165R	MH	Monnier *et al.*, Hum Mutat, 2005		
D166N	MH	Rueffert *et al.*, Acta Anaesthesiol Scand, 2002		
D166G	MH	Robinson *et al.*, Hum Mutat, 2006		
H382N	MH	Broman *et al.*, Br J Anaesth, 2009		
P164S	CPVT	Choi *et al.*, Circulation, 2004		
A165D	CPVT	Xiong *et al.*, J Mol Cell Cardiol, 2018	Molecular dynamics simulations	Xiong *et al.*, J Mol Cell Cardiol, 2018
S166C	Long QT syndrome	Shigemizu *et al.*, PLoS One, 2015		
R169L	CPVT	Ohno *et al.*, PLoS One, 2015		
R169Q	CPVT	Hsueh *et al.*, Int J Cardiol, 2006	Molecular dynamics simulations	Xiong *et al.*, J Mol Cell Cardiol, 2018
			Chemical cross-linking	Present study
G172E	CPVT	Shimamoto *et al.*, Heart, 2022		
E173G	CPVT	Shimamoto *et al.*, Heart, 2022		
R176Q	ARVD2& CPVT	Tiso *et al.*, Hum Mol Genet, 2001	Molecular dynamics simulations	Xiong *et al.*, J Mol Cell Cardiol, 2018
			Chemical cross-linking	Seidel *et al.*, Cardiovasc Res, 2021
G178A	CPVT	Ohno *et al.*, PLoS One, 2015		
D179N	CPVT	Kawata *et al.*, Circ J, 2016		
D400H	SUD	Tester *et al.*, Mayo Clin Proc, 2012		
D401G	SUD	Stattin *et al.*, Int J Legal Med, 2016		

**FIGURE 1:**
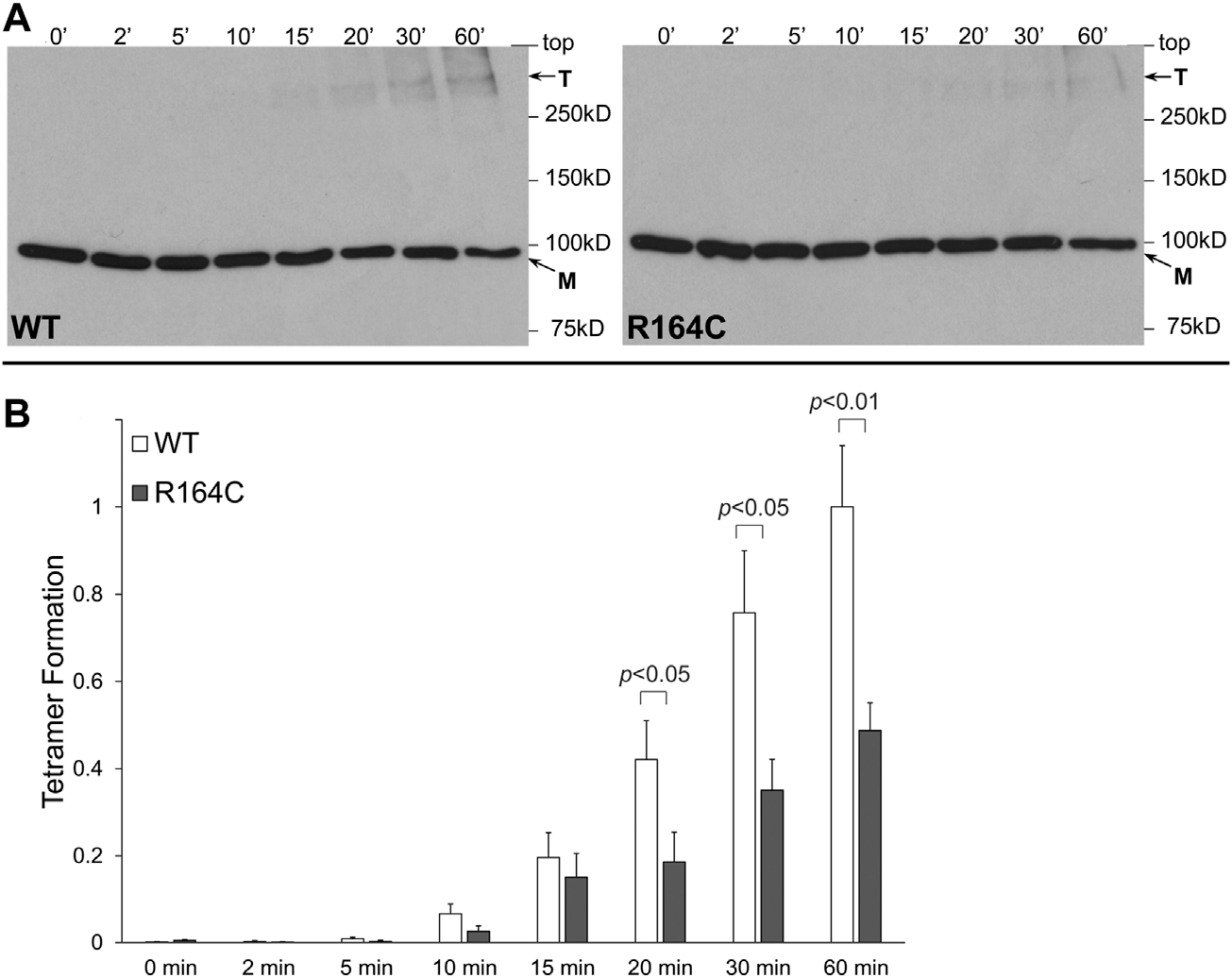
MH mutation R164C disrupts RyR1 NTD tetramerization. Chemical cross-linking assays of HEK293 cell homogenates expressing NT^RyR1−WT^ (rabbit RyR1 residues 1–915, cMyc-tagged) or the R164C mutant, NT^RyR1-R164C^. **(A).** Cell homogenates were incubated with glutaraldehyde for the indicated time points under reducing (10 mM DTT) conditions and analyzed by Western blotting using Ab^cMyc^; monomer (M) and tetramer (T) are indicated with the arrows. **(B).** Densitometry analysis was carried out on the bands corresponding to tetramer and monomer moieties and used to calculate tetramer formation. Data (*n* = 7) are given as mean value ±SEM; statistical analysis was carried out using Mann-Whitney test.

R164C, the corresponding mutation in the rabbit isoform, was introduced by site-directed mutagenesis in rabbit RyR1 N-terminal residues 1–915 tagged with the cMyc peptide epitope (NT^RyR1-R164C^). NT^RyR1−WT^ and NT^RyR1-R164C^ were expressed in mammalian HEK293 cells with an apparent molecular weight of ∼100 kDa as a monomer (Figure 1A). Chemical cross-linking was carried out using glutaraldehyde, a homo-bi-functional reagent with two aldehyde groups that react with the free amino group in the side chain of lysine residues to create stable covalent bonds. Glutaraldehyde does not induce tetramerization but merely cross-links pre-existing tetramers which could then be preserved despite protein denaturation during SDS-PAGE. Cross-linking of NT^RyR1−WT^ and subsequent Western blotting using Ab^cMyc^ revealed the existence of the tetrameric species (Figure 1A), as previously reported (Zissimopoulos et al., 2014). Cross-linking of NT^RyR1-R164C^ also demonstrated the presence of the tetramer, which increased in a time-dependent manner, however its relative abundance was low. Collective data (n = 7) following densitometry analysis indicated that the R164C mutation results in significant reduction (by 51% at 60 min) of the tetramer compared to WT (Figure 1B).

### Rescue of defective RyR2 NTD tetramerization in CPVT

**FIGURE 2:**
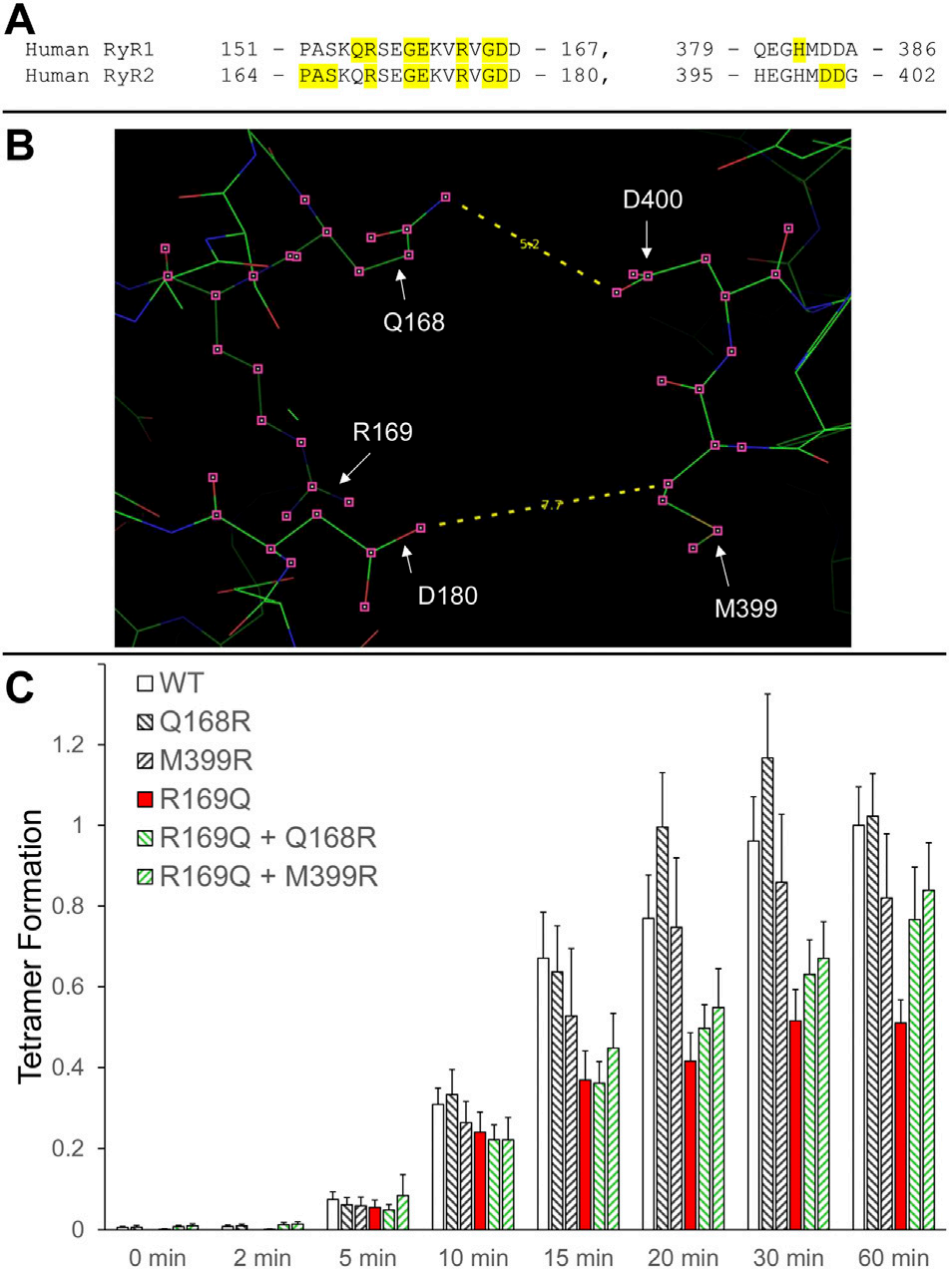
Rescue of defective RyR2 NTD tetramerization in CPVT. **(A).** Peptide sequence of RyR1/2 β8-β9 (left) and β23-β24 loops (right). RyR1 and RyR2 pathogenic mutations are shaded and listed in Table 1. **(B).** Image of the RyR2 N-terminal inter-subunit interface generated using PyMol from the RyR2 closed state (5GO9) structure. The location of the CPVT mutation R169Q, as well as residues Q168 and D180 on one subunit that are in close apposition to residues M399 and D400 on the neighboring subunit, are depicted. The Q168-D400 and D180-M399 distances (in Å) are also indicated. **(C)**. Chemical cross-linking assays of NT^WT^ (human RyR2 residues 1–906, cMyc-tagged), NT^Q168R^, NT^M399R^, NT^R169Q^, NT^R169Q+Q168R^ and NT^R169Q+M399R^ expressed in HEK293 cells. Cell homogenates were incubated with glutaraldehyde for the indicated time points under reducing (10 mM DTT) conditions and analyzed by Western blotting using Ab^cMyc^. Densitometry analysis was carried out on the bands corresponding to tetramer and monomer moieties and used to calculate tetramer formation. Data (n ≥ 7) are given as mean value ±SEM.

We have previously shown that RyR2 NTD tetramerization is mediated by the interaction between the β8-β9 (amino acids 165–179) and β23-β24 loops (amino acids 395–402) (Seidel et al., 2021) (Figure 2A). The experiments described above with RyR1 R163C as well as those with the equivalent residue in RyR2, R176Q (Seidel et al., 2021), demonstrate the involvement of NTD self-association and specifically, of the β8-β9 loop in RyR1/2 pathophysiology. To extend our findings to other β8-β9 loop CPVT mutations (Table 1), we assessed the impact of R169Q on RyR2 NTD tetramerization.

R169Q was introduced by site-directed mutagenesis in human RyR2 N-terminal residues 1–906 tagged with the cMyc peptide epitope (NT^R169Q^). NT^WT^ and NT^R169Q^ were expressed in mammalian HEK293 cells, subjected to chemical cross-linking using glutaraldehyde, and analyzed by Western blotting using Ab^cMyc^. Both NT^WT^ and NT^R169Q^ were capable of tetramer formation but to quantitatively different extents. Collective data (n ≥ 7) following densitometry analysis indicated that the R169Q mutation results in significant reduction (by 49% at 60 min) of the tetramer compared to WT (Figure 2B; Table 2).

**TABLE 2:** Chemical cross-linking assays (n ≥ 7) of RyR2 NT^WT^, NT^Q168R^, NT^M399R^, NT^R169Q^, NT^R169Q+Q168R^ and NT^R169Q+M399R^ as described in the legend to Figure 2. Statistical analysis was carried out for tetramer formation at 60 min of cross-linking using Kruskal–Wallis test with Dunn’s multiple comparison test.

RyR2 NT tetramer formation	Significant?	*p* Value
WT vs. Q168R	ns	>0.9999
WT vs. M399R	ns	>0.9999
WT vs. R169Q	*	0.0161
WT vs. R169Q + Q168R	ns	>0.9999
WT vs. R169Q + M399R	ns	>0.9999
Q168R vs. M399R	ns	>0.9999
Q168R vs. R169Q	*	0.0185
Q168R vs. R169Q + Q168R	ns	>0.9999
Q168R vs. R169Q + M399R	ns	>0.9999
M399R vs. R169Q	ns	>0.9999
M399R vs. R169Q + Q168R	ns	>0.9999
M399R vs. R169Q + M399R	ns	>0.9999
R169Q vs. R169Q + Q168R	ns	>0.9999
R169Q vs. R169Q + M399R	ns	0.4192
R169Q + Q168R vs. R169Q + M399R	ns	>0.9999

We next sought to genetically manipulate the RyR2 peptide sequence in order to normalize NTD tetramerization that was impaired by the R169Q mutation. Candidate amino acids are the ones that form the inter-subunit interface between the β8-β9 and β23-β24 loops, as indicated by the recent high-resolution electron cryo-microscopy structures of RyR1/2 in the closed and open configurations (des Georges et al., 2016; Peng et al., 2016). Specifically, the following pairs of closely apposed residues can be targeted: Q168-D400, D179-M399, D180-H398, D180-M399. We therefore hypothesized that the following two manipulations may reinforce NT^R169Q^ tetramerization (Figure 2B): 1. Substitution of Q168 for arginine, a basic amino acid with a long side chain, which may increase the electrostatic interaction with the negatively-charged side chain of D400, 2. Substitution of M399 for arginine, which may increase the electrostatic interaction with both acidic residues D179 and D180.

Initially, we tested whether Q168R and M399R affect RyR2 NTD tetramerization. As shown in Figure 2C, NT^Q168R^ and NT^M399R^ tetramer formation (102% and 82% of WT at 60 min, respectively) was similar to NT^WT^. We next generated RyR2 NT constructs carrying the CPVT R169Q mutation together with the Q168R or M399R substitution. NT^R169Q+Q168R^ and NT^R169Q+M399R^ displayed higher tetramer formation (77% and 84% of WT at 60 min, respectively) compared to NT^R169Q^ (51% of WT at 60 min). Although NT^R169Q+Q168R^ and NT^R169Q+M399R^ tetramer formation did not reach statistical significance relative to NT^R169Q^, it was also not significantly different to NT^WT^ (Table 2). These results indicate that the engineered Q168R and M399R substitutions partially restore NTD tetramerization to RyR2-R169Q.

## Discussion

Numerous RyR1 and RyR2 mutations associated with MH and CPVT, respectively, have been functionally characterized with the vast majority resulting in gain-of-function channels (Hernandez-Ochoa et al., 2016; Fowler and Zissimopoulos, 2022). However, how pathogenic RyR mutations function at the molecular or structural level, i.e., what is the defective RyR regulatory mechanism(s), is poorly understood. Here, we demonstrate that perturbed NTD tetramerization is a common molecular mechanism operating in RyR1 and RyR2 genetic disease.

RyR1 R163C is one of the most studied MH mutations in heterologous (HEK293) and homologous expression systems (dyspedic myotubes) as well as in heterozygous knockin mice. Results obtained from single channel recordings, [^3^H]ryanodine binding and Ca^2+^ imaging indicate enhanced basal activity, increased sensitivity to Ca^2+^ activation, increased sensitivity to pharmacological activators, and elevated resting cytosolic Ca^2+^ concentration (Tong et al., 1997; Avila and Dirksen, 2001; Yang et al., 2003; Yang et al., 2006; Feng et al., 2011; Chen et al., 2018; Iyer et al., 2020). Here, we used chemical cross-linking to demonstrate that R163C impairs RyR1 NTD tetramerization (Figure 1). Our biochemical findings are in agreement with the 3D structure of RyR1-R163C, which was recently solved at near-atomic resolution under the same closed-state conditions as with RyR1-WT (Iyer et al., 2020). R163C was found to cause a shift and rotation in the NTD that resulted in increased NTD-NTD inter-subunit distance. This stabilized the NTD interaction with the core solenoid, which in turn altered the high-affinity Ca^2+^-binding site. Thus, RyR1-R163C is inherently hyperactive and leaky primarily due to defective N-terminal inter-subunit interactions.

RyR2 R169Q is a gain-of-function CPVT mutation as indicated by higher propensity for spontaneous Ca^2+^ oscillations in HEK293 cells and increased Ca^2+^-dependent [^3^H]ryanodine binding (Nozaki et al., 2020). Our chemical cross-linking analysis demonstrates that R169Q impairs RyR2 NTD tetramerization (Figure 2; Table 2), similar to our previous observation with the R176Q mutation (Seidel et al., 2021). The mutations studied here do not involve the loss or introduction of lysine residues and therefore there is no change in the number or location of amino acids amenable to glutaraldehyde reaction, however, we cannot rule out the possibility that these mutations indirectly alter the ability of glutaraldehyde to cross-link lysine residues in adjacent RyR NT monomers by affecting the local conformation. We should also note that we studied pathogenic mutations in a homozygous scenario, whereas MH and CPVT1 are autosomal dominant diseases. Our results are consistent with previous molecular dynamics simulations indicating that R169Q, as well as A165D and R176Q, increase the NTD-NTD inter-subunit distance (Xiong et al., 2018). Hence, the present study adds to the mounting evidence from biochemical, structural, and molecular dynamics analyses that RyR1 and RyR2 pathogenic mutations located within the β8-β9 loop disrupt NTD tetramerization (Table 1) (Iyer et al., 2020; Seidel et al., 2021; Xiong et al., 2018).

The RyR N-terminus has previously been implicated in neuromuscular and cardiac disease because of disrupted interaction with the central domain (Ikemoto and Yamamoto, 2002), also known as “helical domain-1” (Peng et al., 2016; Yan et al., 2015) and “bridging solenoid (BSol)” (des Georges et al., 2016). However, RyR1-R163C and RyR2-R169Q are unlikely to affect this structure-function parameter because the NTD interface with the BSol (RyR1 residues 2146–2712, RyR2 residues 2111–2679) involves residues other than those of the β8-β9 loop (des Georges et al., 2016; Peng et al., 2016; Yan et al., 2015). Indeed, no differences in the NTD-BSol interface were reported in the RyR1-R163C structure compared to WT (Iyer et al., 2020). On the other hand, altered NTD-BSol interface was recently reported for RyR1-R615C, which induced a distinct pathological conformation in RyR1 to facilitate channel opening (Woll et al., 2021). Thus, N-terminal mutations may affect different RyR inter-domain associations, but all the available evidence suggests that mutations within the β8-β9 loop (e.g., RyR1-R163C, RyR2-R169Q) alter N-terminal inter-subunit interactions.

Importantly, diminished NTD tetramerization due to the CPVT R169Q mutation can be reinforced by introducing positively-charged amino acids at the NTD-NTD interface (Figure 2). R169Q likely results in diminished NTD tetramerization because of the loss of a salt bridge with D400 on the neighboring subunit (Peng et al., 2016; Xiong et al., 2018). On the other hand, our introduced structural mutations Q168R and M399R may create new salt bridges with D400 and D179/D180, respectively, which appear to compensate for the loss of R169-D400 electrostatic interaction. These observations add further weight to the role of the β8-β9 and β23-β24 loops in mediating efficient N-terminal inter-subunit interactions. Notably, they act as proof of concept for stabilizing N-terminal inter-subunit interactions in RyR2 disease. While such genetic manipulation is an unlikely therapeutic tool, the development of small chemical compounds that target the RyR2 NTD-NTD interface holds great promise as potential therapy in CPVT.

## Data Availability

The raw data supporting the conclusion of this article will be made available by the authors, without undue reservation.
